# Bis(μ-biphenyl-2,2′-dicarboxyl­ato)bis­[aqua­(2,2′-bipyridine)cadmium(II)]

**DOI:** 10.1107/S1600536810016387

**Published:** 2010-05-12

**Authors:** Pei-Xi Lin, Yan Cheng, Yu-Lian Mai, Zhe An

**Affiliations:** a School of Chemistry and Life Science, Maoming University, Maoming 525000, People’s Republic of China; b Department of Pharmacy, Mudanjiang Medical University, Mudanjiang, 157011, People’s Republic of China

## Abstract

In the centrosymmetric dinuclear mol­ecule of the title compound, [Cd_2_(C_14_H_8_O_4_)_2_(C_10_H_8_N_2_)_2_(H_2_O)_2_], the Cd^2+^ ion is coordinated by three O atoms from two different diphenyl­dicarboxyl­ate (dpa) ligands (one *O*,*O*′-bidentate and one monodentate), a chelating bipyridine ligand and a water mol­ecule, generating an extremely distorted trigonal-prismatic (or irregular) CdN_2_O_4_ coordination geometry for the metal ion. The bridging ligands generate an 18-membered ring, which is stabilized by two pairs of intra­molecular O—H⋯O hydrogen bonds.

## Related literature

For background to coordination polymers, see: Hagrman *et al.* (1999 ▶); Ghosh & Bharadwaj (2004 ▶); Evans *et al.* (1999 ▶).
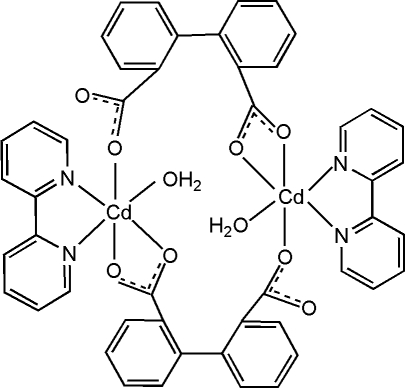


## Experimental

### 

#### Crystal data

[Cd_2_(C_14_H_8_O_4_)_2_(C_10_H_8_N_2_)_2_(H_2_O)_2_]
*M* *_r_* = 1053.61
Monoclinic, 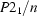

*a* = 11.532 (2) Å
*b* = 10.961 (2) Å
*c* = 16.891 (3) Å
β = 98.37 (3)°
*V* = 2112.4 (7) Å^3^
*Z* = 2
Mo *K*α radiation
μ = 1.07 mm^−1^
*T* = 295 K
0.12 × 0.10 × 0.08 mm

#### Data collection

Bruker APEXII CCD diffractometer
Absorption correction: multi-scan (*SADABS*; Bruker, 2001 ▶) *T*_min_ = 0.882, *T*_max_ = 0.919
15936 measured reflections
3697 independent reflections
3223 reflections with *I* > 2σ(*I*)
*R*_int_ = 0.030

#### Refinement

*R*[*F* ^2^ > 2σ(*F* ^2^)] = 0.025
*wR*(*F* ^2^) = 0.063
*S* = 1.00
3697 reflections
295 parameters
H atoms treated by a mixture of independent and constrained refinement
Δρ_max_ = 0.59 e Å^−3^
Δρ_min_ = −0.26 e Å^−3^

### 

Data collection: *APEX2* (Bruker, 2004 ▶); cell refinement: *SAINT-Plus* (Bruker, 2001 ▶); data reduction: *SAINT-Plus*; program(s) used to solve structure: *SHELXS97* (Sheldrick, 2008 ▶); program(s) used to refine structure: *SHELXL97* (Sheldrick, 2008 ▶); molecular graphics: *SHELXTL* (Sheldrick, 2008 ▶); software used to prepare material for publication: *SHELXTL*.

## Supplementary Material

Crystal structure: contains datablocks I, global. DOI: 10.1107/S1600536810016387/hb5426sup1.cif

Structure factors: contains datablocks I. DOI: 10.1107/S1600536810016387/hb5426Isup2.hkl

Additional supplementary materials:  crystallographic information; 3D view; checkCIF report

## Figures and Tables

**Table 1:** Selected bond lengths (Å)

Cd1—O4	2.1960 (18)
Cd1—O1	2.2540 (18)
Cd1—N2	2.324 (2)
Cd1—N1	2.362 (2)
Cd1—O5	2.385 (2)
Cd1—O2	2.586 (2)

**Table 2:** Hydrogen-bond geometry (Å, °)

*D*—H⋯*A*	*D*—H	H⋯*A*	*D*⋯*A*	*D*—H⋯*A*
O5—H1*W*⋯O4^i^	0.81 (4)	1.94 (4)	2.738 (3)	168 (4)
O5—H2*W*⋯O2^i^	0.80 (4)	2.28 (4)	2.932 (3)	138 (3)

Symmetry code: (i) 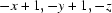.

**Fig. 1.**
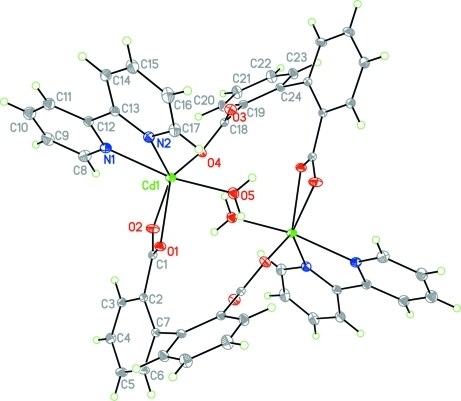
The molecular structure of (I), drawn with 30% probability displacement ellipsoids for the non-hydrogen atoms. Unlablled atoms are generated by (1–x, 1–y, –z).

## supplementary crystallographic information

### Comment

The design of inorganic-organic supramolecular complexes has received long-lasting research interest not only because of their appealing structural and topological novelty but also due to their unusual optical, electronic, magnetic and catalytic properties, and their further potential medical value derived from their antiviral and the inhibition of angiogenesis (Hagrman *et al.*, 1999; Ghosh *et al.*, 2004; Evans *et al.*, 1999). In this paper, we report one new metal complexes constructed from 2,2-bipyridine, diphenate, and cadmium(II) ion.

Figure 1 gives the Cd atom is coordinated by three oxygen atoms from two different dpa ligands with Cd—O bond distance range from 2.1964 (19) to 2.586 (2) %A, and two nitrogen atoms from one bipyridine ligand (average Cd—N distance 2.343 %A). Two such asymmetric units connect to form an 18-numbered ring, which contains two Cd atoms, two dpa ligands, and two bipyridine ligands.

### Experimental

A mixture of cadmium(II) acetate (1 mmol), diphenic acid (1 mmol), 2,2'-bipyridine (1 mmol), sodium hydroxide (2 mmol)and water (15 ml) was stirred for 30 min in air. The mixture was then transferred to a 25 ml Teflon-lined hydrothermal bomb. The bomb was kept at 433 K for 72 h under autogenous pressure. Upon cooling, colorless prisms of (I) were obtained from the reaction mixture.

### Refinement

The water H atoms were located in a difference map and freely refined. All C-bound H atoms were placed in calculated positions with C—H = 0.93Å and refined as riding with U_iso_(H) = 1.2U_eq_(carrier).

### Crystal data

**Table:**

[Cd_2_(C_14_H_8_O_4_)_2_(C_10_H_8_N_2_)_2_(H_2_O)_2_]	*F*(000) = 1056
*M**_r_* = 1053.61	*D*_x_ = 1.656 Mg m^−^^3^
Monoclinic, *P*2_1_/*n*	Mo *K*α radiation, λ = 0.71073 Å
Hall symbol: -P 2yn	Cell parameters from 3697 reflections
*a* = 11.532 (2) Å	θ = 3.1–25.0°
*b* = 10.961 (2) Å	µ = 1.07 mm^−^^1^
*c* = 16.891 (3) Å	*T* = 295 K
β = 98.37 (3)°	Block, colorless
*V* = 2112.4 (7) Å^3^	0.12 × 0.10 × 0.08 mm
*Z* = 2	

### Data collection

**Table:**

Bruker APEXII CCD diffractometer	3697 independent reflections
Radiation source: fine-focus sealed tube	3223 reflections with *I* > 2σ(*I*)
graphite	*R*_int_ = 0.030
phi and ω scans	θ_max_ = 25.0°, θ_min_ = 3.1°
Absorption correction: multi-scan (*SADABS*; Bruker, 2001)	*h* = −13→13
*T*_min_ = 0.882, *T*_max_ = 0.919	*k* = −12→13
15936 measured reflections	*l* = −20→20

### Refinement

**Table:**

Refinement on *F*^2^	Primary atom site location: structure-invariant direct methods
Least-squares matrix: full	Secondary atom site location: difference Fourier map
*R*[*F*^2^ > 2σ(*F*^2^)] = 0.025	Hydrogen site location: inferred from neighbouring sites
*wR*(*F*^2^) = 0.063	H atoms treated by a mixture of independent and constrained refinement
*S* = 1.00	*w* = 1/[σ^2^(*F*_o_^2^) + (0.0345*P*)^2^ + 0.8848*P*] where *P* = (*F*_o_^2^ + 2*F*_c_^2^)/3
3697 reflections	(Δ/σ)_max_ = 0.003
295 parameters	Δρ_max_ = 0.59 e Å^−^^3^
0 restraints	Δρ_min_ = −0.26 e Å^−^^3^

### Special details

**Table:**

Geometry. All esds (except the esd in the dihedral angle between two l.s. planes) are estimated using the full covariance matrix. The cell esds are taken into account individually in the estimation of esds in distances, angles and torsion angles; correlations between esds in cell parameters are only used when they are defined by crystal symmetry. An approximate (isotropic) treatment of cell esds is used for estimating esds involving l.s. planes.
Refinement. Refinement of *F*^2^ against ALL reflections. The weighted *R*-factor wR and goodness of fit *S* are based on *F*^2^, conventional *R*-factors *R* are based on *F*, with *F* set to zero for negative *F*^2^. The threshold expression of *F*^2^ > σ(*F*^2^) is used only for calculating *R*-factors(gt) etc. and is not relevant to the choice of reflections for refinement. *R*-factors based on *F*^2^ are statistically about twice as large as those based on *F*, and *R*- factors based on ALL data will be even larger.

### Fractional atomic coordinates and isotropic or equivalent isotropic displacement parameters (Å^2^)

**Table:**

	*x*	*y*	*z*	*U*_iso_*/*U*_eq_	
C1	0.7107 (2)	0.6689 (2)	−0.01343 (15)	0.0395 (6)	
C2	0.8213 (2)	0.6527 (2)	−0.04980 (14)	0.0346 (5)	
C3	0.9266 (2)	0.6536 (2)	0.00182 (15)	0.0432 (6)	
H3	0.9251	0.6607	0.0565	0.052*	
C4	1.0330 (2)	0.6445 (3)	−0.02535 (17)	0.0531 (7)	
H4	1.1025	0.6464	0.0103	0.064*	
C5	1.0349 (2)	0.6323 (3)	−0.10596 (18)	0.0616 (8)	
H5	1.1062	0.6257	−0.1253	0.074*	
C6	0.9320 (2)	0.6300 (3)	−0.15806 (16)	0.0534 (7)	
H6	0.9351	0.6218	−0.2125	0.064*	
C7	0.8229 (2)	0.6394 (2)	−0.13230 (14)	0.0375 (5)	
C8	0.6572 (3)	0.8714 (3)	0.17640 (18)	0.0637 (8)	
H8	0.7079	0.8055	0.1759	0.076*	
C9	0.6904 (3)	0.9662 (4)	0.22807 (19)	0.0726 (10)	
H9	0.7603	0.9631	0.2633	0.087*	
C10	0.6179 (3)	1.0650 (3)	0.2263 (2)	0.0730 (9)	
H10	0.6388	1.1311	0.2599	0.088*	
C11	0.5144 (3)	1.0669 (3)	0.17499 (19)	0.0603 (8)	
H11	0.4654	1.1346	0.1724	0.072*	
C12	0.4840 (2)	0.9658 (2)	0.12683 (15)	0.0434 (6)	
C13	0.3711 (2)	0.9582 (2)	0.07259 (16)	0.0415 (6)	
C14	0.2774 (3)	1.0359 (2)	0.0786 (2)	0.0586 (8)	
H14	0.2833	1.0947	0.1187	0.070*	
C15	0.1764 (3)	1.0254 (3)	0.0250 (2)	0.0705 (9)	
H15	0.1133	1.0771	0.0284	0.085*	
C16	0.1693 (3)	0.9385 (3)	−0.0332 (2)	0.0696 (9)	
H16	0.1022	0.9311	−0.0708	0.084*	
C17	0.2630 (3)	0.8622 (3)	−0.03524 (19)	0.0573 (7)	
H17	0.2575	0.8022	−0.0745	0.069*	
C18	0.3844 (2)	0.5407 (2)	0.13566 (14)	0.0378 (5)	
C19	0.3754 (2)	0.4450 (2)	0.19899 (14)	0.0373 (5)	
C20	0.4655 (2)	0.4387 (3)	0.26274 (15)	0.0495 (7)	
H20	0.5271	0.4940	0.2653	0.059*	
C21	0.4666 (3)	0.3531 (3)	0.32235 (17)	0.0628 (8)	
H21	0.5282	0.3508	0.3645	0.075*	
C22	0.3765 (3)	0.2714 (3)	0.31919 (18)	0.0635 (9)	
H22	0.3767	0.2127	0.3589	0.076*	
C23	0.2850 (3)	0.2767 (3)	0.25660 (17)	0.0519 (7)	
H23	0.2236	0.2213	0.2552	0.062*	
C24	0.2821 (2)	0.3629 (2)	0.19543 (14)	0.0383 (5)	
Cd1	0.495536 (15)	0.710476 (16)	0.035750 (10)	0.03941 (8)	
N1	0.5558 (2)	0.8693 (2)	0.12699 (13)	0.0474 (5)	
N2	0.36180 (19)	0.87021 (19)	0.01676 (13)	0.0437 (5)	
O1	0.62029 (16)	0.71087 (16)	−0.05533 (11)	0.0462 (4)	
O2	0.71202 (18)	0.6405 (2)	0.05848 (11)	0.0609 (5)	
O3	0.29828 (17)	0.58894 (17)	0.09796 (11)	0.0519 (5)	
O4	0.49025 (16)	0.56627 (16)	0.12540 (11)	0.0494 (4)	
O5	0.37320 (18)	0.60995 (18)	−0.07047 (13)	0.0514 (5)	
H1W	0.417 (3)	0.566 (3)	−0.091 (2)	0.080*	
H2W	0.330 (3)	0.563 (3)	−0.053 (2)	0.080*	

### Atomic displacement parameters (Å^2^)

**Table:**

	*U*^11^	*U*^22^	*U*^33^	*U*^12^	*U*^13^	*U*^23^
C1	0.0421 (15)	0.0358 (12)	0.0425 (15)	−0.0027 (11)	0.0129 (12)	−0.0079 (11)
C2	0.0344 (13)	0.0329 (12)	0.0377 (13)	−0.0020 (10)	0.0088 (10)	−0.0028 (10)
C3	0.0428 (15)	0.0466 (14)	0.0400 (14)	−0.0024 (12)	0.0056 (11)	−0.0080 (12)
C4	0.0339 (15)	0.0671 (19)	0.0553 (17)	−0.0017 (13)	−0.0031 (12)	−0.0098 (15)
C5	0.0313 (15)	0.094 (2)	0.0616 (18)	−0.0063 (15)	0.0133 (13)	−0.0190 (18)
C6	0.0382 (15)	0.082 (2)	0.0422 (14)	−0.0055 (14)	0.0144 (12)	−0.0101 (15)
C7	0.0350 (13)	0.0397 (13)	0.0385 (13)	−0.0046 (11)	0.0084 (10)	−0.0027 (11)
C8	0.0521 (19)	0.078 (2)	0.0586 (18)	0.0112 (16)	−0.0011 (15)	−0.0007 (17)
C9	0.056 (2)	0.102 (3)	0.0561 (19)	−0.014 (2)	−0.0035 (15)	−0.0080 (19)
C10	0.076 (2)	0.073 (2)	0.070 (2)	−0.016 (2)	0.0110 (19)	−0.0173 (18)
C11	0.063 (2)	0.0493 (16)	0.070 (2)	−0.0067 (14)	0.0148 (16)	−0.0082 (15)
C12	0.0482 (16)	0.0409 (14)	0.0443 (14)	−0.0029 (12)	0.0174 (12)	0.0052 (12)
C13	0.0433 (15)	0.0298 (12)	0.0543 (15)	0.0016 (11)	0.0168 (12)	0.0089 (12)
C14	0.056 (2)	0.0354 (14)	0.087 (2)	0.0062 (13)	0.0204 (17)	−0.0016 (14)
C15	0.0437 (19)	0.0454 (17)	0.122 (3)	0.0111 (14)	0.0120 (18)	0.0057 (19)
C16	0.0488 (19)	0.0480 (17)	0.106 (3)	0.0099 (14)	−0.0086 (17)	0.0095 (18)
C17	0.0501 (18)	0.0490 (16)	0.0695 (19)	0.0062 (14)	−0.0026 (15)	0.0035 (15)
C18	0.0421 (15)	0.0355 (12)	0.0373 (13)	−0.0017 (11)	0.0103 (11)	−0.0066 (11)
C19	0.0373 (14)	0.0421 (13)	0.0329 (12)	−0.0012 (11)	0.0066 (10)	−0.0024 (11)
C20	0.0454 (16)	0.0560 (16)	0.0446 (15)	−0.0121 (13)	−0.0019 (12)	0.0008 (13)
C21	0.064 (2)	0.072 (2)	0.0460 (16)	−0.0134 (17)	−0.0120 (14)	0.0123 (16)
C22	0.075 (2)	0.070 (2)	0.0425 (16)	−0.0123 (17)	−0.0016 (15)	0.0205 (15)
C23	0.0537 (18)	0.0588 (17)	0.0431 (15)	−0.0147 (14)	0.0068 (13)	0.0064 (13)
C24	0.0354 (13)	0.0469 (14)	0.0339 (12)	−0.0030 (11)	0.0093 (10)	−0.0009 (11)
Cd1	0.03751 (12)	0.04095 (12)	0.04107 (12)	0.00950 (8)	0.01014 (8)	0.00319 (8)
N1	0.0439 (13)	0.0520 (13)	0.0469 (12)	0.0065 (11)	0.0083 (10)	0.0021 (11)
N2	0.0412 (12)	0.0378 (11)	0.0520 (13)	0.0066 (9)	0.0073 (10)	0.0055 (10)
O1	0.0362 (10)	0.0566 (11)	0.0475 (10)	0.0040 (8)	0.0119 (8)	−0.0030 (9)
O2	0.0582 (13)	0.0849 (15)	0.0443 (11)	0.0056 (11)	0.0225 (9)	0.0077 (11)
O3	0.0480 (12)	0.0506 (11)	0.0569 (11)	0.0086 (9)	0.0069 (9)	0.0115 (9)
O4	0.0416 (11)	0.0511 (10)	0.0575 (11)	−0.0050 (9)	0.0132 (9)	0.0107 (9)
O5	0.0435 (12)	0.0496 (12)	0.0609 (13)	−0.0003 (9)	0.0073 (9)	0.0015 (10)

### Geometric parameters (Å, °)

**Table:**

C1—O2	1.252 (3)	C15—C16	1.363 (5)
C1—O1	1.259 (3)	C15—H15	0.9300
C1—C2	1.505 (3)	C16—C17	1.370 (4)
C2—C3	1.388 (3)	C16—H16	0.9300
C2—C7	1.404 (3)	C17—N2	1.337 (4)
C3—C4	1.375 (3)	C17—H17	0.9300
C3—H3	0.9300	C18—O3	1.219 (3)
C4—C5	1.371 (4)	C18—O4	1.289 (3)
C4—H4	0.9300	C18—C19	1.513 (3)
C5—C6	1.371 (4)	C19—C20	1.385 (4)
C5—H5	0.9300	C19—C24	1.397 (3)
C6—C7	1.394 (3)	C20—C21	1.375 (4)
C6—H6	0.9300	C20—H20	0.9300
C7—C24^i^	1.493 (3)	C21—C22	1.367 (4)
C8—N1	1.334 (4)	C21—H21	0.9300
C8—C9	1.375 (5)	C22—C23	1.382 (4)
C8—H8	0.9300	C22—H22	0.9300
C9—C10	1.366 (5)	C23—C24	1.397 (4)
C9—H9	0.9300	C23—H23	0.9300
C10—C11	1.369 (5)	C24—C7^i^	1.493 (3)
C10—H10	0.9300	Cd1—O4	2.1960 (18)
C11—C12	1.389 (4)	Cd1—O1	2.2540 (18)
C11—H11	0.9300	Cd1—N2	2.324 (2)
C12—N1	1.343 (3)	Cd1—N1	2.362 (2)
C12—C13	1.481 (4)	Cd1—O5	2.385 (2)
C13—N2	1.342 (3)	Cd1—O2	2.586 (2)
C13—C14	1.391 (4)	O5—H1W	0.81 (4)
C14—C15	1.372 (5)	O5—H2W	0.80 (4)
C14—H14	0.9300		
			
O2—C1—O1	121.9 (2)	C16—C17—H17	118.5
O2—C1—C2	118.4 (2)	O3—C18—O4	123.4 (2)
O1—C1—C2	119.7 (2)	O3—C18—C19	122.4 (2)
C3—C2—C7	119.2 (2)	O4—C18—C19	114.2 (2)
C3—C2—C1	117.3 (2)	C20—C19—C24	119.2 (2)
C7—C2—C1	123.5 (2)	C20—C19—C18	117.6 (2)
C4—C3—C2	122.1 (2)	C24—C19—C18	123.2 (2)
C4—C3—H3	119.0	C21—C20—C19	122.0 (3)
C2—C3—H3	119.0	C21—C20—H20	119.0
C5—C4—C3	118.8 (3)	C19—C20—H20	119.0
C5—C4—H4	120.6	C22—C21—C20	119.5 (3)
C3—C4—H4	120.6	C22—C21—H21	120.3
C6—C5—C4	120.1 (2)	C20—C21—H21	120.3
C6—C5—H5	119.9	C21—C22—C23	119.6 (3)
C4—C5—H5	119.9	C21—C22—H22	120.2
C5—C6—C7	122.3 (2)	C23—C22—H22	120.2
C5—C6—H6	118.8	C22—C23—C24	121.8 (3)
C7—C6—H6	118.8	C22—C23—H23	119.1
C6—C7—C2	117.4 (2)	C24—C23—H23	119.1
C6—C7—C24^i^	116.8 (2)	C23—C24—C19	117.9 (2)
C2—C7—C24^i^	125.8 (2)	C23—C24—C7^i^	116.5 (2)
N1—C8—C9	123.2 (3)	C19—C24—C7^i^	125.5 (2)
N1—C8—H8	118.4	O4—Cd1—O1	123.82 (7)
C9—C8—H8	118.4	O4—Cd1—N2	123.57 (7)
C10—C9—C8	118.2 (3)	O1—Cd1—N2	112.39 (7)
C10—C9—H9	120.9	O4—Cd1—N1	96.65 (8)
C8—C9—H9	120.9	O1—Cd1—N1	106.70 (7)
C9—C10—C11	120.0 (3)	N2—Cd1—N1	70.19 (8)
C9—C10—H10	120.0	O4—Cd1—O5	96.51 (7)
C11—C10—H10	120.0	O1—Cd1—O5	81.60 (7)
C10—C11—C12	118.7 (3)	N2—Cd1—O5	86.34 (8)
C10—C11—H11	120.6	N1—Cd1—O5	156.53 (7)
C12—C11—H11	120.6	O4—Cd1—O2	78.88 (7)
N1—C12—C11	121.6 (3)	O1—Cd1—O2	53.40 (6)
N1—C12—C13	116.3 (2)	N2—Cd1—O2	148.28 (7)
C11—C12—C13	122.1 (3)	N1—Cd1—O2	86.32 (8)
N2—C13—C14	120.5 (3)	O5—Cd1—O2	115.29 (7)
N2—C13—C12	116.7 (2)	C8—N1—C12	118.2 (3)
C14—C13—C12	122.8 (3)	C8—N1—Cd1	124.6 (2)
C15—C14—C13	119.7 (3)	C12—N1—Cd1	117.16 (18)
C15—C14—H14	120.2	C17—N2—C13	118.8 (2)
C13—C14—H14	120.2	C17—N2—Cd1	121.77 (18)
C16—C15—C14	119.4 (3)	C13—N2—Cd1	117.42 (17)
C16—C15—H15	120.3	C1—O1—Cd1	100.02 (15)
C14—C15—H15	120.3	C1—O2—Cd1	84.66 (16)
C15—C16—C17	118.7 (3)	C18—O4—Cd1	111.79 (16)
C15—C16—H16	120.7	Cd1—O5—H1W	105 (3)
C17—C16—H16	120.7	Cd1—O5—H2W	110 (3)
N2—C17—C16	122.9 (3)	H1W—O5—H2W	104 (4)
N2—C17—H17	118.5		

Symmetry codes: (i) −*x*+1, −*y*+1, −*z*.

### Hydrogen-bond geometry (Å, °)

**Table:**

*D*—H···*A*	*D*—H	H···*A*	*D*···*A*	*D*—H···*A*
O5—H1W···O4^i^	0.81 (4)	1.94 (4)	2.738 (3)	168 (4)
O5—H2W···O2^i^	0.80 (4)	2.28 (4)	2.932 (3)	138 (3)

Symmetry codes: (i) −*x*+1, −*y*+1, −*z*.
